# F- and G-Actin Concentrations in Lamellipodia of Moving Cells

**DOI:** 10.1371/journal.pone.0004810

**Published:** 2009-03-11

**Authors:** Stefan A. Koestler, Klemens Rottner, Frank Lai, Jennifer Block, Marlene Vinzenz, J. Victor Small

**Affiliations:** 1 Institute of Molecular Biotechnology, Austrian Academy of Sciences, Vienna, Austria; 2 Cytoskeleton Dynamics Group, Helmholtz Centre for Infection Research (HZI), Braunschweig, Germany; University of Birmingham, United Kingdom

## Abstract

Cells protrude by polymerizing monomeric (G) into polymeric (F) actin at the tip of the lamellipodium. Actin filaments are depolymerized towards the rear of the lamellipodium in a treadmilling process, thereby supplementing a G-actin pool for a new round of polymerization. In this scenario the concentrations of F- and G-actin are principal parameters, but have hitherto not been directly determined. By comparing fluorescence intensities of bleached and unbleached regions of lamellipodia in B16-F1 mouse melanoma cells expressing EGFP-actin, before and after extraction with Triton X-100, we show that the ratio of F- to G-actin is 3.2+/−0.9. Using electron microscopy to determine the F-actin content, this ratio translates into F- and G-actin concentrations in lamellipodia of approximately 500 µM and 150 µM, respectively. The excess of G-actin, at several orders of magnitude above the critical concentrations at filament ends indicates that the polymerization rate is not limited by diffusion and is tightly controlled by polymerization/depolymerization modulators.

## Introduction

Eukaryotic cells move by the extension of a leaf-like structure, the lamellipodium, at the cell front [1]. Protrusion occurs by polymerization of actin filaments at the tip of the lamellipodium, thereby pushing the membrane forward [2]. Actin filaments are polar, with the barbed, fast growing ends pointing towards the direction of protrusion [3]. Under steady state conditions the network of actin filaments in lamellipodia maintains a constant breadth by coordinated depolymerization from the filament pointed ends towards the rear, in a treadmilling regime [2], [4]–[6]. Treadmilling relies in the first instance on inherent differences of critical concentration for growth at the two filament ends, measured in vitro as around 0.06 µM and 0.6 µM at the plus and minus ends, respectively [7], [8]. Regulation can take place on several levels: actin filament nucleation, elongation and depolymerization, monomer sequestration and filament end capping [8], [9]. For an understanding of the basic principles of actin turnover and for simulating the molecular scenarios underlying protrusion [10] the biochemical parameters in vivo and, not least, the concentrations of F- and G-actin in the lamellipodium need to be established.

Global estimates of F- and G-actin ratios obtained by the fractionation of cell extracts [11]–[15] and the use of the DNAse I inhibition assay [16] showed that there are approximately equivalent amounts of polymerized and unpolymerized actin in non-muscle cells, with estimates of the monomeric actin concentration ranging widely, from 12–300 µM [7]. Only recently were techniques developed to directly quantify the local concentrations of proteins in living cells, namely in fission yeast. In a careful, fluorescence-based approach [17] obtained global concentrations by quantitative immunoblotting and local concentrations from the relative fluorescence intensity. The relative concentrations of F- and G-actin were not however addressed. Estimates of actin filament concentrations in lamellipodia range from 700 µM, based on filament counts from electron microscopy [18] to 1600 µM, derived from the comparison of the phalloidin label intensities of single filaments and lamellipodia of fixed cells [19]. The latter authors concluded that the G-actin concentration at the lamellipodium tip was in the range of 8 µM, based on *in vitro* rate constants for polymerization [19].

In this work we established a method to determine the F- and G-actin concentrations in the lamellipodium. Our measurements demonstrate a local concentration of G-actin in lamellipodia of around 150 µM, several orders of magnitude higher than the critical concentration for polymerization.

## Results and Discussion

### Concentration of F-actin in lamellipodia

Our estimates of F–actin concentration are based on counts of filament numbers in aldehyde/Triton fixed and negatively-stained lamellipodia [20]. By monitoring the extraction/fixation process during the preparation of cells for electron microscopy in the light microscope we have shown that the gradient of intensity of EGFP-actin across lamellipodia can be preserved by our fixation protocol [20]. This gradient correlates with a progressive drop in filament number away from the front of the lamellipodium that we suppose reflects a graded length of filaments with all plus ends located near the tip. The location of filament plus ends at the tip is consistent with the restriction of the WAVE nucleation complex to the actin-membrane interface [21]; Supplemental figure S1 and Supplemental text S1).

A complementation of previous filament counts close to the front edge of the lamellipodium of B16-F1 melanoma cells [20], yielded a value of 103 filaments per µm (sdm = 17; 20 measurements in 5 cells) in continuously protruding lamellipodia segments (Supplemental figure S2 and Supplemental text S1). Calculation of the concentration of F-actin requires a value for the thickness of the lamellipodium. Various methods have been used to estimate the thickness of lamellipodia, including thin section electron microscopy [1], standing wave fluorescence microscopy [19], stereo microscopy of negatively stained preparations [18] and atomic force microscopy, with values ranging from around 70–180 nm. Plastic cross sections of B16 cell lamellipodia showed a constant thickness across their breadth that varied between 70 and 100 nm (not shown). Taking into account some shrinkage during embedding and other published estimates, we assume here a lamellipodia thickness in B16-F1 cells of 120 nm. Future measurements by cryo electron tomography will lead to a more accurate estimate of this value. Taking this thickness and a density of about 100 filaments/µm at the front of the lamellipodium the concentration of F-actin was estimated as roughly 500 µM (for the calculation see Materials and Methods).

### Monomeric EGFP-actin recovers rapidly in bleached lamellipodia

**Figure 1:**
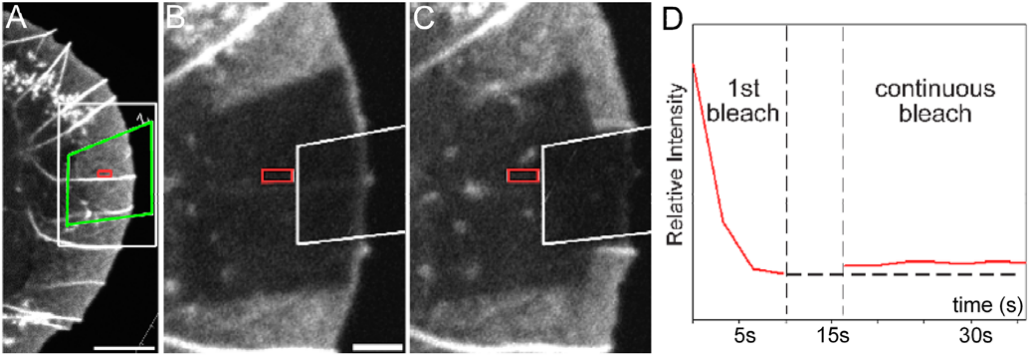
Dual bleach experiments demonstrate that monomeric GFP-actin recovers in the lamellipodium within 6 s after photobleach and is incorporated into F-actin only at the tip. A shows the overview before bleach. Bar, 5 µm. B, C, Enlarged images of the region indicated by the white box in A show the bleached region immediately (B) and 20 s after bleach (C). Bar, 3 µm. Following the first bleach of the lamellipodium (green box in A), bleaching was continued in the region outlined by the box in B and C. Graph (D) shows the average intensity, measured in the red rectangular region, over time. The time between the two vertical dashed lines corresponds to the switch period between the end of the initial bleach and the initiation of the second bleach. Note that the intensity in the region marked by the red square stays constant during the second (continuous) bleach of the more distal region.

To measure the G-actin component in lamellipodia we took advantage of the spatial features of recovery of EGFP-actin fluorescence after photobleaching (Figure 1). During the early phase of recovery of F-actin fluorescence at the lamellipodium front, the rest of the bleached zone is populated by monomeric EGFP-actin. The fluorescence signal in this zone should reflect the G-actin concentration given that it recovers before incorporation of F-actin from the front. To estimate the rate of recovery of the EGFP-G-actin component in the body of the lamellipodium, we performed a double bleach experiment, in which bleaching of the lamellipodium was followed by selective bleaching at the tip (Figure 1). In this way we were able to determine the EGFP-actin signal in the lamellipodium in the absence of recovery of fluorescence at the tip. Within the limits of sensitivity of the double-headed confocal microscope system employed in this experimental setting, the EGFP fluorescence intensity in the lamellipodium was already recovered by the time of the first image acquisition after the initial photobleach (within 6 s; period indicated by dashed lines in Figure 1). The EGFP fluorescence intensity in the bleached zone in the early recovery after photobleach could then be taken as a concentration indicator. The competence of the expressed EGFP-actin to incorporate into filaments was tested by treating cells with jasplakinolide. At concentrations of 100 nM jasplakinolide EGFP-actin fluorescence became concentrated in a progressively distorted lamellipodium region, with a concomittent loss of fluorescence in the lamella zone (not shown), indicating that most, if not all of the expressed EGFP-actin was polymerisation competent.

### Selective extraction of the G-actin component

**Figure 2:**
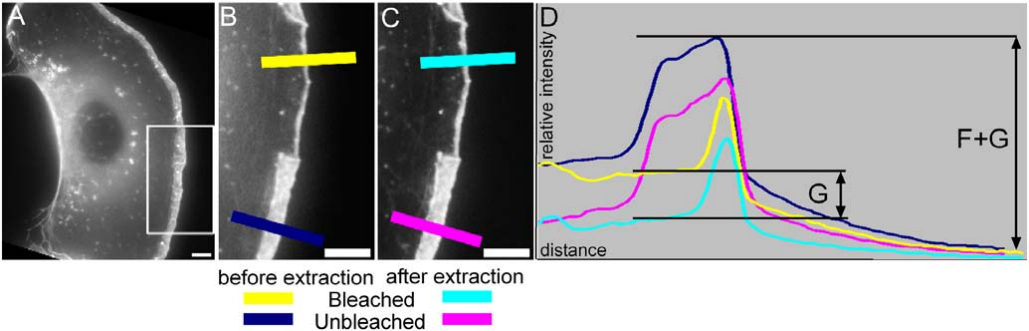
Selective extraction of G-actin after photobleaching. A) Overview of a EGFP-actin expressing cell just before photobleach. B, C: Enlarged region indicated in A) about 2 s after bleach (B) and after extraction (C). Bars, 5 µm. Graph shows intensity scans along the lines indicated in B and C. Dark blue line: unbleached region before extraction; yellow: bleached region before extraction; pink: unbleached region after extraction; light blue: bleached region after extraction. Note the preservation of the intensity gradient in the unbleached region before and after extraction.

**Figure 3:**
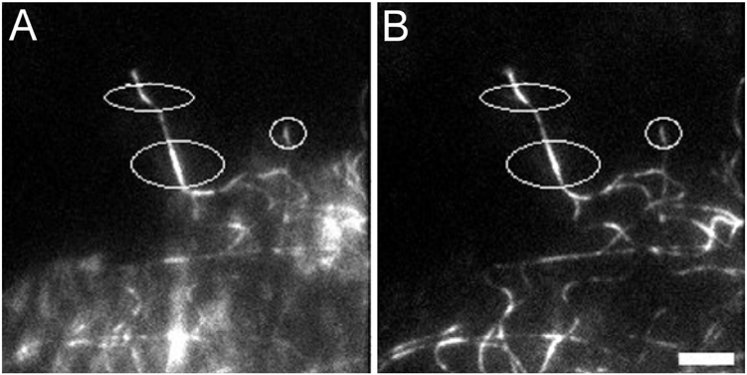
The fluorescence intensity of EGFP does not change upon cell-lysis in PEG-buffer. In order to be able to correlate the EGFP-intensities in live and lysed cells, intensity measurements of EGFP-α-tubulin in B16-F1 cells were carried out before (A) and after (B) addition of detergent. In contrast to actin filaments, microtubules can be imaged as single polymers by fluorescence microscopy, thereby making it feasible to measure the intensity of the same fibres before and after extraction. To reduce the influence of the soluble fraction of EGFP-tubulin on the intensity measurements, microtubules in the periphery and close to the substrate were chosen and imaged by TIRF microscopy. To avoid chemical fixatives, PEG was used to stabilize the cytoskeleton. The difference in maximum fluorescence values of microtubules before and after extraction was around 3.9% (mean, standard deviation = 3.0%, 33 measurements in 14 cells). Since this difference is negligible no correction was applied to the experimental data for actin. Ellipses indicate regions for measurements.

Estimates of the amount of monomeric actin in lamellipodia were obtained by extracting cells with Triton X-100 during the early phase of recovery after photobleach and measuring the drop in fluorescence in the bleached zone (Figure 2). The extraction conditions had then to satisfy two criteria to justify attribution of the loss of fluorescence to monomeric EGFP-actin: 1, F-actin should be mainly retained in the cytoskeleton; and 2, the change in conditions (pre- versus post-extraction) should not affect the fluorescence characteristics of EGFP (or the magnitude of the change should be known). Experiments showed that while the Triton/glutaraldehyde mixture used for electron microscopy satisfied the first criterion, the presence of glutaraldehyde caused a gradual quenching of the EGFP signal. Other extraction conditions were therefore investigated. By using polyethylene glycol in the extraction mixture (see Materials and Methods) without glutaraldehyde, both conditions could be closely satisfied. First, the tip to rear gradient of EGFP-actin fluorescence in the unbleached regions of the lamellipodium could be preserved, indicating retention of the main component of F-actin (Figure 2). As an additional precaution we chose to use bleached regions of lamellipodia for our measurements since we could then avoid any errors due to possible losses of F-actin during extraction. Second, the fluorescence intensity of single microtubules in B16 cells transfected with EGFP-tubulin, measured by TIRF microscopy, before and after applying the extraction protocol, was essentially unchanged (Figure 3).

In the representative extraction experiment shown in Figure 2 bleaching was performed using a confocal scanning head and image acquisition pre- and post-extraction with a CCD camera for optimal sensitivity (see Materials and Methods; [6]. Taking the fluorescence intensity at the unbleached lamellipodium tip before extraction as a measure of F-+G-actin, we obtained an average F- to G-actin ratio of 3.2∶1 (SDM = 0.86, n = 11). The experimental values for the cells used for analysis are shown in supplemental table S1. With a concentration of F-actin from the filament counts of 490 µM (see above), this ratio translates a G-actin concentration of around 150 µM. Here we assumed that the G-actin concentration at the tip of the lamellipodium is similar to that a few µm (between 1 and 3 µm) behind, where the measurements in the bleached regions were made. This assumption was consistent with the more or less constant thickness of the lamellipodium (in EM cross sections, not shown) and the level drop of fluorescence intensity across the lamellipodium upon lysis (Figure 2).

Our estimates indicate that the G-actin concentration in the lamellipodium is about 1500 times higher than the critical concentration required for elongation at the barbed end. Based on in vitro rate constants [7] this concentration would support polymerization rates up to 260 µm/min! Therefore the concentration of actin in lamellipodia is itself not a limiting factor for protrusion. A funnelling mechanism of actin assembly in the lamellipodium, whereby G-actin is limiting and capping protein blocks a subpopulation of barbed filament ends so that the remaining uncapped filaments can grow faster [8] is difficult to reconcile with such a high monomer concentration. From recent modelling of G-actin mobility and consumption in lamellipodia [10] entertain one scenario whereby G-actin depletion could contribute to shape changes in keratocyte lamellipodia. Our finding of a 15-fold higher concentration in lamellipodia than that assumed by Novak and colleagues [19] suggests that other regulatory factors are responsible for modulating lamellipodia form.

How is the high G-actin concentration within the lamellipodium maintained? Observations by Zicha et al. [22] in T15 rat fibroblasts suggested that the transport of G-actin into lamellipodia occurred faster than could be explained by diffusion. Rapid transport from the lamella region into the lamellipodium was also confirmed by Lai et al. [6]. Zicha et al. [22] suggested that G-actin diffusion is supplemented by myosin dependent contraction of the cell body. This certainly could occur as a result of retraction induced spreading [23], [24]. However, in B16-F1 cells, inhibition of myosin II by blebbistatin does not affect the transport of photoactivated GFP-actin from the lamella to the lamellipodium tip (Block and Rottner, unpublished data), and causes a transient increase of the protrusion rate [20], strongly suggesting myosin II-independent mechanisms in this cell type. We cannot however exclude a role of unconventional myosins in potentiating the delivery of monomeric actin complexes to the tips of lamellipodia and filopodia.

In conclusion, these first direct estimates of the F- and G-actin concentrations in lamellipodia of living cells provide basic parameters for the further development of ideas about the mode of protrusion and the regulation of actin polymerization and depolymerisation during cell migration.

## Materials and Methods

The conditions for transfection and imaging B16-F1 cells were as previously described [20].

Single FRAP experiments were performed using an LSM 510 Meta (Zeiss, Jena, Germany) confocal head for bleaching and an interline transfer, progressive scan CCD camera (Coolsnap_HQ_; Photometrics, Tucson, AZ, USA; or Cascade II, Roper Scientific) driven by Metamorph software (Molecular Devices Corp., Downingtown, PA, USA) for acquisition [6]. Imaging was performed with a 100×1.45NA αPlan-FLUAR TIRF objective (Zeiss). Selected cellular areas covering parts of protruding lamellipodia were bleached (20–30 iterations at full laser power at 488 nm, 30 mW argon laser). Immediately after one full-frame scan of respective fields, imaging was switched to epi-fluorescence, with a mercury lamp (100 W) as light source. Switching time was approximately 2 s.

Dual-bleach experiments were performed using a double-scan-headed confocal microscope (Fluoview1000, Olympus), allowing simultaneous imaging (with 30 mW 488 nm multiline argon at laser power of approximately 1–5%) and photobleaching using a 20 mW 405 nm diode laser. Output laser power was approximately 5–10% for photobleaching. A 100×1.45NA PlanApo TIRF objective (Olympus Inc.) was used in all experiments. Movies were acquired at a scanning rate of 2.711 or 3.264 s per frame. The initial photobleach of the region covering the whole breadth of the lamellipodium was followed by programmed initiation of a second movie and continuous bleaching of a new, distal region of the lamellipodium. The instrumental switching time between the initial and the second bleach periods resulted in a gap in image acquisition of approximately 6 seconds. Image analysis was carried out on a PC using FV10-ASW 1.6 viewer (Olympus Inc., Olympus, Hamburg, Germany) and Metamorph (Molecular Devices Corp.) software.

For lysis, cells were observed in 4% polyethylene glycol (20.000 g/mol) in cytoskeleton buffer (see e.g. [20]) without EGTA and prepared with pipes (pH 7.0). Triton X-100 was added from a 20% stock to a final concentration of 1% within 5 s after photobleaching. EGFP-actin was purchased from Clontech (Mountain View, CA, USA).

The actin concentrations were calculated from the mean value of the actin filament number, 103 per µm, and the F∶G-actin ratio of 3.2∶1: Assuming continuous filaments, the total length of filaments in a 1×1 µm sheet is 103×10^3^ nm. The volume in 1 µm^2^ of Lamellipodium with 120 nm thickness corresponds to 1.2×10^−10^ µl. Taking 13 subunits per 38 nm of filament length, the number of actin molecules in a 1×1 µm sheet gives 35.24×10^3^ molecules in 1.2×10^−10^ µl. This makes 2.94×10^20^ molecules per litre, corresponding to an F-actin concentration of 488 µM.

EGFP-α-tubulin (mouse) expressing cells were observed with a Zeiss Axiovert 200 equipped for TIRF microscopy (Zeiss/Visitron) and with a 100×1.45NA αPlan-FLUAR TIRF objective (Zeiss), solid state 488 nm laser, and a Cascade camera (Roper Scientific), and lysed as above including 1 µM Taxol to stabilize microtubules. Analysis was performed on a Windows PC with Metamorph Software.

## Supporting Information

Figure S1 Correlative light- and electron microscopy and immunogold labeling of EGFP-Abi1. Note localization of 10 nm gold label (black dots) at the tip of the cell edge. The definition of the filaments is reduced compared to Supplemental figure 2 because of the treatment for immunogold labelling. Bar, 200 nm. Inset shows the living EGFP-Abi1 expressing cell in the light microscope just before fixation. The rectangle indicates the region of the electron micrograph. Bar, 10 µm. For Materials and Methods see supplemental text S1).
(3.65 MB TIF)

Figure S2 Correlative light- and electron microscopy of the lamellipodium of an EGFP-actin expressing B16-F1 cell. The single filaments are well defined. The image was processed with the bandpass filter in ImageJ. Bar, 200 nm. Inset shows the living cell just before fixation. The rectangle indicates the region of the electron micrograph. Bar, 10 µm. For Material and Methods see supplemental text S1.
(5.58 MB TIF)

Table S1 Intensity data and calculations of F- to G-actin in B16 cell lamellipodia. Data for cells 1–9 was obtained with a Coolsnap, and for cells 10 and 11 with a Cascade camera. The positions for measurements of the different values are indicated in Figure 2.
(0.07 MB DOC)

Text S1 Online supplemental material and methods: Correlative light and electron microscopy and Immunolabelling of B16-F1 cells.
(0.03 MB DOC)
