# Supplementary figures and images for: F- and G-Actin Concentrations in Lamellipodia of Moving Cells
Source: PLoS One. 2009 Mar 11;4(3):e4810. doi: 10.1371/journal.pone.0004810 (PMC2652108; doi:10.1371/journal.pone.0004810)

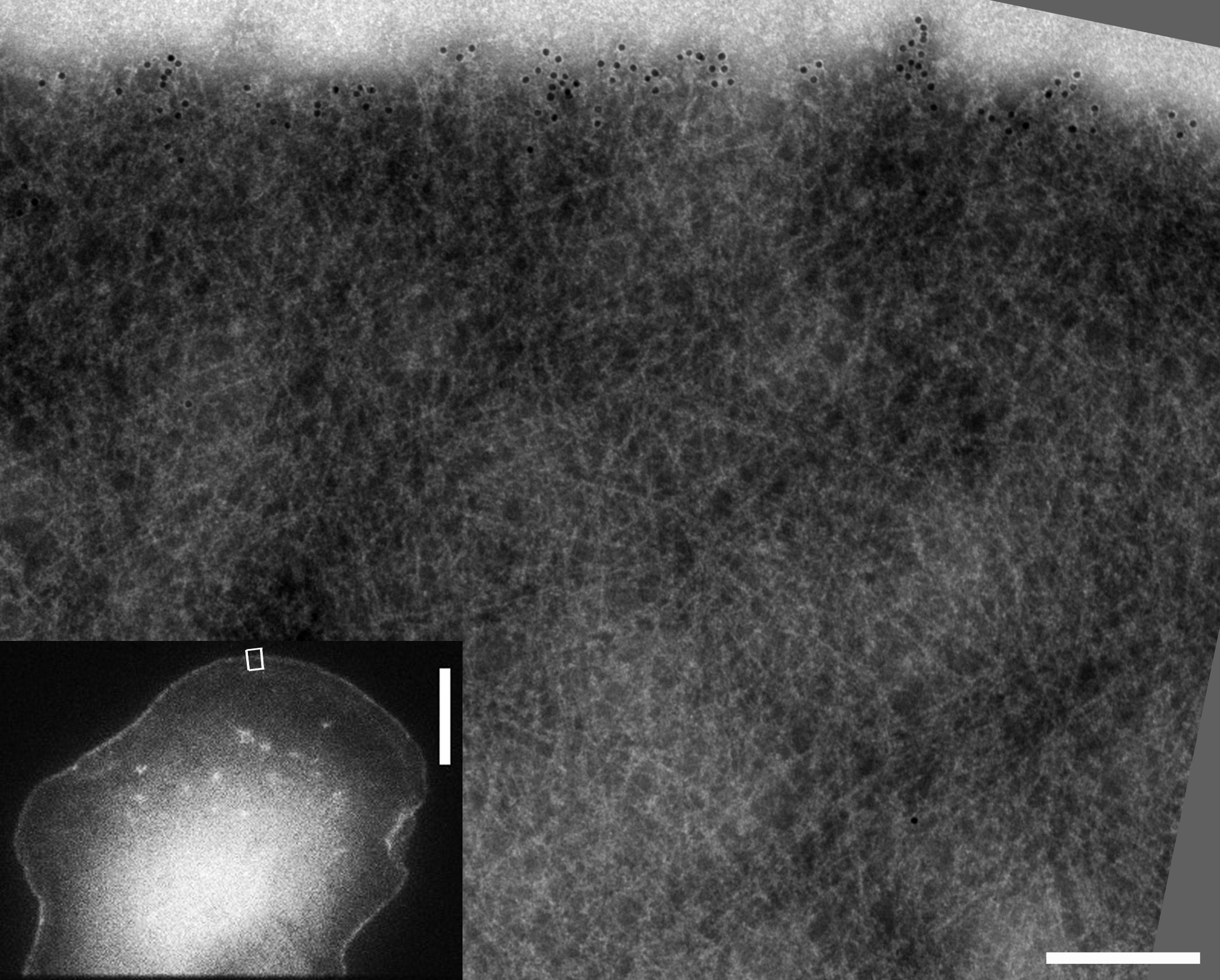

Supplement: Figure S1 — Correlative light- and electron microscopy and immunogold labeling of EGFP-Abi1. Note localization of 10 nm gold label (black dots) at the tip of the cell edge. The definition of the filaments is reduced compared to Supplemental figure 2 because of the treatment for immunogold labelling. Bar, 200 nm. Inset shows the living EGFP-Abi1 expressing cell in the light microscope just before fixation. The rectangle indicates the region of the electron micrograph. Bar, 10 µm. For Materials and Methods see supplemental text S1). (3.65 MB TIF) [file pone.0004810.s001.tif]

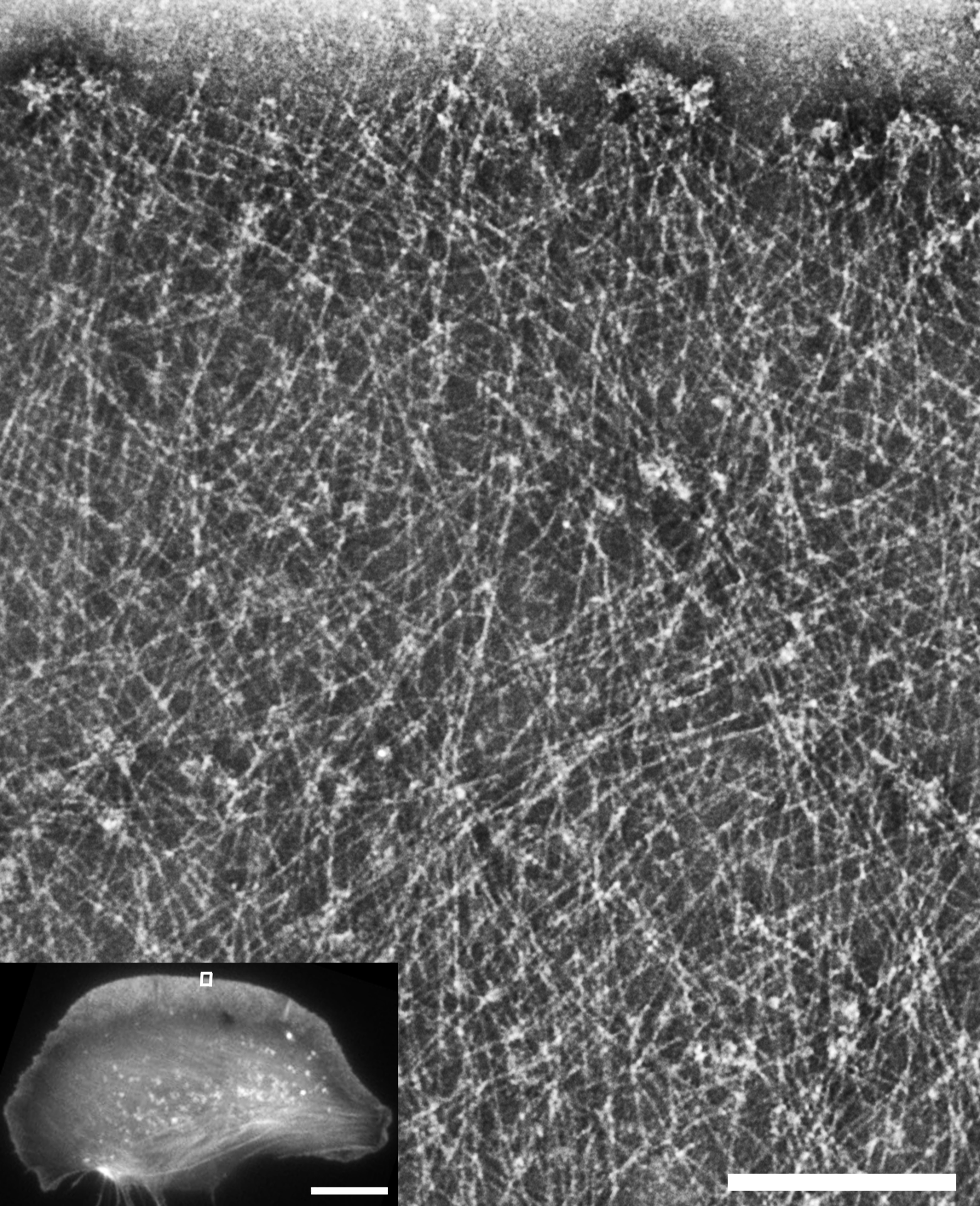

Supplement: Figure S2 — Correlative light- and electron microscopy of the lamellipodium of an EGFP-actin expressing B16-F1 cell. The single filaments are well defined. The image was processed with the bandpass filter in ImageJ. Bar, 200 nm. Inset shows the living cell just before fixation. The rectangle indicates the region of the electron micrograph. Bar, 10 µm. For Material and Methods see supplemental text S1. (5.58 MB TIF) [file pone.0004810.s002.tif]
