# Supplementary material for: F- and G-Actin Concentrations in Lamellipodia of Moving Cells
Source: PLoS One. 2009 Mar 11;4(3):e4810. doi: 10.1371/journal.pone.0004810 (PMC2652108; doi:10.1371/journal.pone.0004810)
Supplement: Table S1 — Intensity data and calculations of F- to G-actin in B16 cell lamellipodia. Data for cells 1–9 was obtained with a Coolsnap, and for cells 10 and 11 with a Cascade camera. The positions for measurements of the different values are indicated in Figure 2. (0.07 MB DOC) [file pone.0004810.s003.doc]

| **cell** |  | **unbleached** | **bleached** | **background** | **F+G** | **G** | **F** | **F:G** |
| --- | --- | --- | --- | --- | --- | --- | --- | --- |
|  |  | Intensity measurements | | | Pre-extraction unbleached  minus  background | Pre-extraction unbleached  minus  post-extraction unbleached | F+G  minus  G | F/G |
| 1 | pre-extraction | 345 | 189 | 107 | 238 | 45 | 193 | 4,3 |
|  | post-extraction | 316 | 144 |  |  |  |  |  |
| 2 | pre-extraction | 206 | 159 | 108 | 98 | 28 | 70 | 2,5 |
|  | post-extraction | 186 | 131 |  |  |  |  |  |
| 3 | pre-extraction | 227 | 169 | 109 | 118 | 29 | 89 | 3,1 |
|  | post-extraction | 206 | 140 |  |  |  |  |  |
| 4 | pre-extraction | 257 | 198,5 | 99 | 158 | 50 | 108 | 2,2 |
|  | post-extraction | 215 | 148,5 |  |  |  |  |  |
| 5 | pre-extraction | 156 | 127 | 104 | 52 | 18 | 34 | 1,9 |
|  | post-extraction | 139 | 109 |  |  |  |  |  |
| 6 | pre-extraction | 181 | 133 | 99 | 82 | 15 | 67 | 4,5 |
|  | post-extraction | 167 | 118 |  |  |  |  |  |
| 7 | pre-extraction | 359 | 201,5 | 114 | 245 | 54,5 | 190,5 | 3,5 |
|  | post-extraction | 310 | 147 |  |  |  |  |  |
| 8 | pre-extraction | 144 | 118,5 | 96 | 48 | 10,5 | 37,5 | 3,6 |
|  | post-extraction | 137 | 108 |  |  |  |  |  |
| 9 | pre-extraction | 179 | 131 | 96 | 83 | 19,5 | 63,5 | 3,3 |
|  | post-extraction | 160 | 111,5 |  |  |  |  |  |
| 10 | pre-extraction | 25387 | 11782 | 2111 | 23276 | 4674 | 18602 | 4 |
|  | post-extraction | 21223 | 7108 |  |  |  |  |  |
| 11 | pre-extraction | 24581 | 13195 | 2742 | 21839 | 6030 | 15809 | 2,6 |
|  | post-extraction | 21220 | 7165 |  |  |  |  |  |
|  |  |  |  |  |  |  | average | 3,2 |
|  |  |  |  |  |  |  | stdev | 0,9 |
