# Supplementary material for: F- and G-Actin Concentrations in Lamellipodia of Moving Cells
Source: PLoS One. 2009 Mar 11;4(3):e4810. doi: 10.1371/journal.pone.0004810 (PMC2652108; doi:10.1371/journal.pone.0004810)
Supplement: Text S1 — Online supplemental material and methods: Correlative light and electron microscopy and Immunolabelling of B16-F1 cells. (0.03 MB DOC) [file pone.0004810.s004.doc]

**Supplemental Text S1: Online supplemental material and methods**

EGFP-Abi1 [25] and mCherry-actin [20] were made as described. Correlative light- and electron-microscopy was performed as described [20] on EGFP-actin or EGFP-Abi1 and mCherry-actin transfected B16-F1 cells. EGFP-Abi1 expressing cells were treated with AlF for 15 – 30 min before and during observation with the light microscope. For immunogold labelling, this protocol was modified in the following way: After fixation under the light microscope the cells were incubated with anti-EGFP-antibodies (rabbit; kindly provided by Dr. Jan Faix, Hannover) in PBS containing 1 µg/ml phalloidin for two days, washed with PBS and followed by incubation with 10 nm gold-conjugated anti-rabbit-antibodies (BBInternational, Cardiff, UK) for two days.
